# Feasibility of Classifying Life Stages and Searching for the Determinants: Results from the Medical Expenditure Panel Survey 1996–2011

**DOI:** 10.3389/fpubh.2017.00247

**Published:** 2017-10-27

**Authors:** Yi-Sheng CHAO, Hau-tieng Wu, Chao-Jung Wu

**Affiliations:** ^1^Centre de recherche du centre hospitalier de l’Université de Montréal (CRCHUM), Université de Montréal, Montréal, QC, Canada; ^2^Department of Mathematics, University of Toronto, Toronto, ON, Canada; ^3^Mathematics Division, National Center for Theoretical Sciences, Taipei, Taiwan; ^4^Département d’Informatique, UQAM, Montréal, QC, Canada

**Keywords:** life stages, principal components, principal component analysis, medical expenditure panel survey, stages of transition, stable stages

## Abstract

**Background:**

Life stages are not clearly defined and significant determinants for the identification of stages are not discussed. This study aims to test a data-driven approach to define stages and to identify the major determinants.

**Methods:**

This study analyzed the data on the Medical Expenditure Panel Survey interviewees from 1996 to 2011 in the United States. This study first selected features with the Spearman’s correlation to remove redundant variables and to increase computational feasibility. The retained 430 variables were log transformed, if applicable. Sixty-four nominal variables were replaced with 164 binominal variables. This led to 525 variables that were available for principal component analysis (PCA). Life stages were proposed to be periods of ages with significantly different values of principal components (PCs).

**Results:**

After retaining subjects followed throughout the panels, 244,089 were eligible for PCA, and the number of civilians was estimated to be 4.6 billion. The age ranged from 0 to 90 years old (mean = 35.88, 95% CI = 35.67–36.09). The values of the first PC were not significant from age of 6 to 13, 30 to 41, 46 to 60, and 76 to 90 years (adjusted *p* > 0.5), and the major determinants were related to functional status, employment, and poverty.

**Conclusion:**

Important stages and their major determinants, including the status of functionality and cognition, income, and marital status, can be identified. Identifying stages of stability or transition will be important for research that relies on a research population with similar characteristics to draw samples for observation or intervention.

**Contribution:**

This study sets an example of defining stages of transition and stability across ages with social and health data. Among all available variables, cognitive limitations, income, and poverty are important determinants of these stages.

## Introduction

Life course perspective links exposures in early life to incidence in later life and has been proven useful to understand distant courses of particular events, such as cardiovascular diseases and diabetes (1, 2). For example, life course epidemiology attempts to find the associations between childhood trajectories and the outcomes in later life (2, 3). Although similar ideas have been widely applied and embedded in terms like adolescence (4) and adulthood (1), it is still unclear how stages that consist of a life course could be clearly defined (1). There are theories on the transitions or trajectories of life (5), some of which are supported by evidence, to show that physiological functions evolve with different developmental stages (6, 7) and health trajectories differ at the end of life (8–10). However, these definitions might not be suitable for general questions, such as what is the beginning of aging (11) and what characteristics can be used to define healthy aging and trajectories? These questions are usually complicated by socioeconomical activities. Therefore, a population perspective is inevitable, and explicit criteria to extract information from population data should be established, if we would like to study the diverse nature of life course.

To address the need to have a better understanding of life course from a population perspective and to establish criteria for data extraction, we consider the Medical Expenditure Panel Survey (MEPS) database that documents several distinctive dimensions of life course, especially socioeconomic status, functionality, and health status. The MEPS is a source of information with important characteristics, especially national representativeness and age coverage from 0 to 90 years (12). The main theme of the MEPS includes health insurance coverage, health-care consumption, and incurred expenditures (12). It also contains a wide range of individual information on demographics, income and tax filing, health status, disability, access to care, employment, health insurance and health utilization with information on expenditure and source of payment (12). For health status, detailed information is collected on activities of daily living, instrumental activities of daily living, vision, hearing, changes in limitations, child health and preventive care, and other dimensions measured by SF-12, smoking status, Kessler Index (K6), Patient Health Questionnaire, and attitudes about health (12). Due to its rich set of variables on individual status, national coverage, and longitudinal components, the MEPS data are not only used in the health expenditure estimation but also in other social research topics, such as income tax simulations (13, 14), employment, social determinants of health (15), and longitudinal research on individual or family behaviors (12). The inclusion and balance of social and health dimensions in the MEPS database is one of the best resources available for us to establish explicit criteria to understand population data from a life course perspective.

This study aims to test the feasibility of a data-driven approach to classify potential life stages and search for determinants of the components with data on health and health-care utilization. First, we attempt to identify representative components of the population data based on applying a commonly used data summary method, linear or ordinary principal component analysis (PCA). Second, we search for life stages within the principal components (PCs) of the database. Finally, we interpret the identified life stages with the variables that are highly associated with them.

## Data and Methods

The data were explored by PCA and represented by PCs. In this work, the PCs were taken as representative components or *leading trajectories* in a life course perspective in this exploratory project. The leading PCs might represent distinctive dimensions of life course, since they were found to be composed of distinctive input variables. A *life stage* is defined to be a period of consecutive ages, during which the chosen leading trajectories has a small variation. The chronological ages between life stages were *stages of transition*. The entire analytical process is shown step by step below.

### Data Sets

This study analyzed the 16 longitudinal panels released from the MEPS that were conducted annually among the civilian non-institutionalized population to produce nationally representative statistics since 1996 in the United States (16). Each panel lasted for 2 years and consisted of five rounds of data collection (17).

### Data Linkage and Processing

The 16 longitudinal panels of the MEPS were pooled and merged by variable names common to all panels. There were 1,989 common variables across 16 panels (for panels beginning throughout 1996 and 2011, see Datasheet 1 in Supplementary Material for the list of variables and their characteristics). Only subjects participating throughout the 2-year panels were retained in the data set, in addition to those deceased after a 1-year follow-up and before the end of the 2-year panels. Administrative variables and the variables that were used to flag certain circumstances in the process of data gathering were not used for analysis. The result was that only the 789 variables containing individual information in the first years of the 2-year panels were eligible for analysis.

Reserved values that identified specific responses across all variables were recoded according to the MEPS codebooks: −2 recoded to the same answers in previous rounds, −1 to inapplicability and others to missing values (−3, −7, −8, and −9 for “no data in round,” “refused,” “do not know,” and “not ascertained,” respectively; see Datasheet 1 in Supplementary Material for the proportions of these categories in the variable list). The proportions of missingness ranged from 0 to 23.62% (median 0.01% among 644 variables with any missing values, see Datasheet 1 in Supplementary Material for the proportions of missingness). Missing values in all variables were imputed with the multivariate imputation by chained equations (18). The skewness of each continuous variable was evaluated from the raw data, without adjusting the survey design. Log transformation was applied, if the skewness of a log-transformed variable was less than that of the original variable (19).

#### Feature Selection with Spearman’s Rank-Order Correlation

This study first selected features with a correlation-based method proposed for the purpose of removing redundant variables and increasing computational feasibility (20, 21). The data redundancy might be created for the ease of survey implementation or data labeling. For example, different sources of income were asked about separately, and total income was the sum of incomes from all sources (22). The levels of education might be presented as years spent in school or types of highest grades completed (22) (see Datasheet 1 in Supplementary Material for details on variable names and labels).

**Figure 1:**
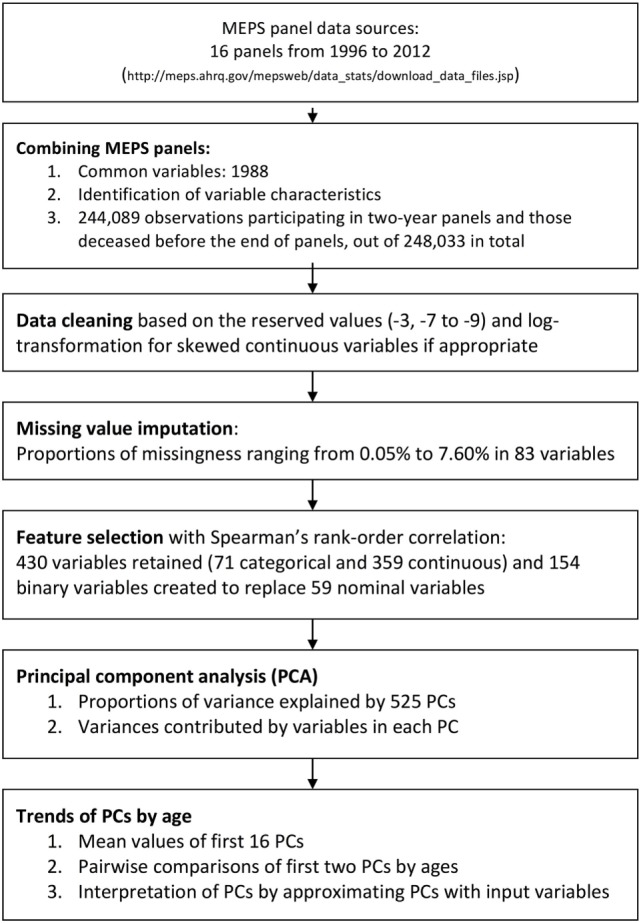
Flowchart of data linkage, data processing, and feature selection with the Medical Expenditure Panel Survey (MEPS) 1996–2011.

First, sex and race/ethnicity were excluded, since they do not provide dynamical information. Age is also excluded, so that we could examine the life stage without the influence of the physical status. Spearman’s rank-order correlation was used to create a correlation matrix of all variables, categorical, or continuous (20, 21). The threshold for redundancy was Spearman’s rank correlation coefficient greater than 0.9 (23). There were 430 variables left for further analysis (see Figure 1 for the flowchart).

Of the 71 categorical variables, 12 ordinal variables that ranked poverty categories, difficulty in using fingers to grasp, self-rated health status, and self-rated mental health status and a summary measure of vision impairment were not transformed to dummy variables. Another 59 nominal variables were replaced with 154 multiple binominal variables. This results in 525 variables that were available for PCA. There were an additional 15 variables that were used for personal identification and control for survey design.

#### Principal Component Analysis

Principal component analysis was proven to be useful for dimension reduction or data preprocessing (24). We considered linear or ordinary PCA as the optimal and most feasible option in consideration of complex survey design (25). Although there are different variations of PCA (24, 26, 27) or similar data techniques (28, 29), there were limited choices of dimension reduction methods under the complex survey design (30). Before PCA, each variable was centered to 0 and scaled to unit variance. The PC comes from projecting input variables to the determined principal vector. The leading PCs had the largest variances and explained the largest proportions of total variances in a database. In this study, PCA was conducted with the 525 variables, while adjusting for complex survey design (30).

The contributions of variable variance to each PC were calculated in two steps. First, for each PC, the associated squared loadings of all input variables were obtained. These squared loadings ranged from 0 to 1. The *contribution of the ith input variable* in each PC is then defined as the variance of each PC was multiplied by the *i*th squared values.

#### Proposed Life Stages

The proposed life stage is evaluated from the first or second PC. We partition the dataset into subsets according to age, ranging from 0 to 90 years old. The age is determined on December 31 of each year. Each *age subset* consists of subjects with the same age, and there are a total of 91 age subsets. We view the *p* value < 0.05 as statistical significant, and the *p* values were adjusted for multiple comparisons with the Benjamini–Hochberg method (31). We call a group of consecutive age subsets *stable*, if less than 5% of all pairs of age subsets are different with statistical significance. In practice, the life stages were searched in the following greedy manner. We began with the age 0 subset and tested whether the PC value at that age subset was significantly different from the age one subset. If there was no significant difference, we found a stable group consisting of age 0 and 1 subset. We continued the iteration, and supposed that we find a stable group consisting of age 0 to age *i* subsets, where *i* > 1. We then determined if the group that consisted of age 0 to age *i* + 1 subsets is a stable group. This search stops when the age *j*, where *j* > *i*, does not belong to the stable group consisting of age 0 to age *j* − 1 subsets. We then restart the greedy iteration from the age *j* subset and view the stable group consisting of age 0 to age *j* − 1 subsets as an isolated group. This greedy algorithm ends when the 91 age subsets are exhausted. We call the period of consecutive ages in a stable group longer than 5 years a *life stage*. The ages between any two life stages were considered stages of transition.

### PC Approximation and Interpretation

For the purpose of interpretation, the PCs were approximated with input variables using a linear regression model. The approximation method assessed the relative importance of all input variables in terms of *R*^2^ regarding each PC. We applied the forward selection to include input variables in the linear regression model. There were no limits on the number of input variables that could be used to approximate each PC. The PCs were interpreted according to the regression coefficients of the input variables.

This study adopted R (v. 3.20 released in April 2015) and R Studio (v. 0.99.441 released in May 2015) for data analysis. The complex survey design of the MEPS was accounted for with the *survey* package (30), except for Spearman’s rank-order correlation and multiple imputations that required more computational capacity than what we could afford. The *R*^2^ used to select the input variables for PC interpretation was assessed with the *relaimpo* package (32).

## Results

### Population Characteristics

**Figure 2:**
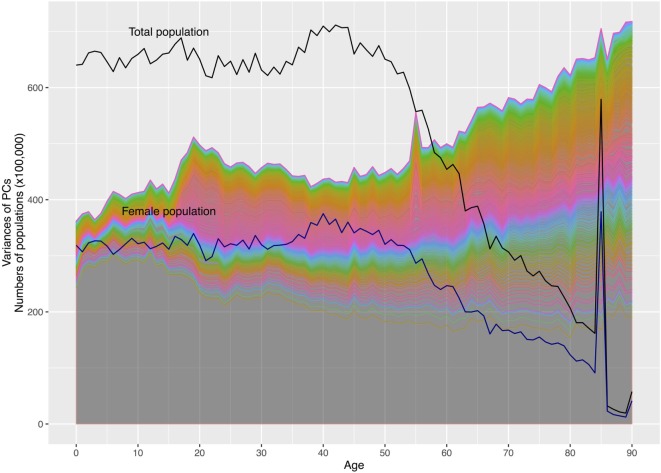
The numbers of population and variances of principal components (PCs) by age. Note: the area under the first lowest curve represents the variance of the first principal component (PC1); the area between the first and second lowest curves represents the variance of the second PC (PC2); this principle applies to other areas under curves. There are 525 PCs in the graph. The upper black solid line represents the numbers of total populations by age; the lower represents the numbers of female populations. The spikes of population numbers at age 85 are due to the top censoring of age beginning in 2000.

**Figure 3:**
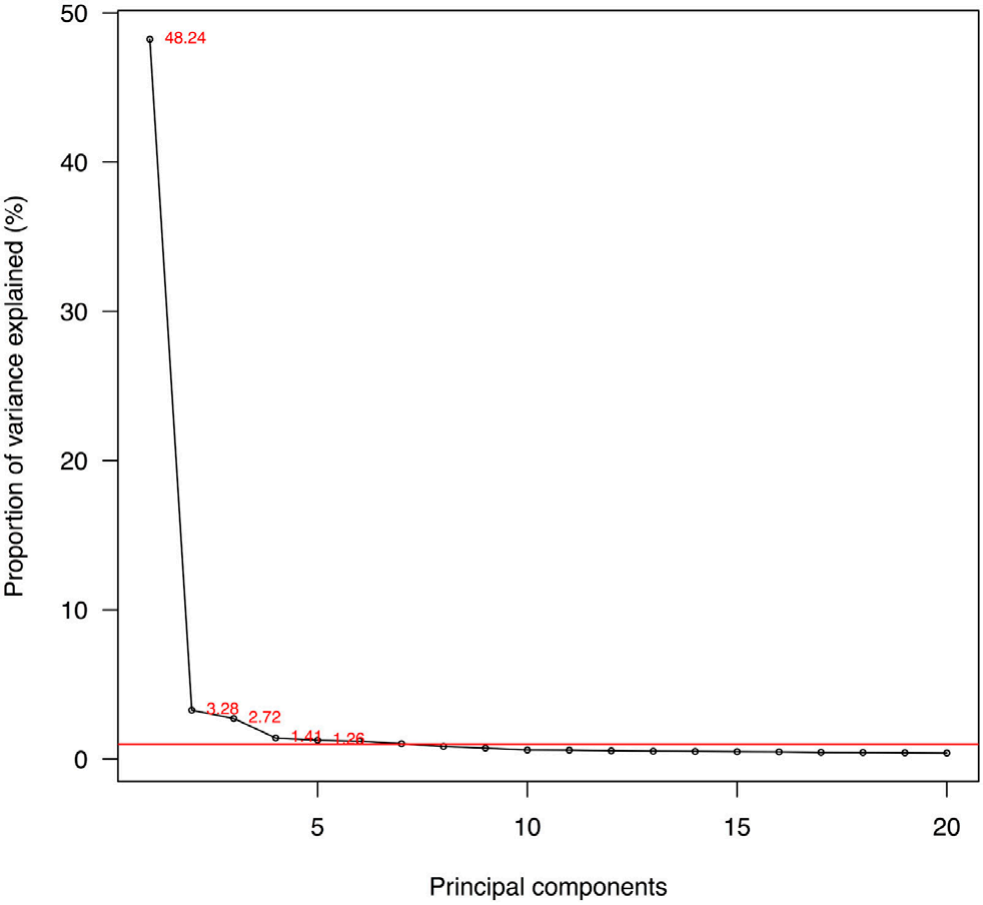
Proportions of variances explained by the first 20 principal components (PCs). Note: red line: 1% of total variance; 525 PCs in total.

There were 248,033 subjects available in panels 1–16 of the MEPS between 1996 and 2011 in the United States. After retaining those participating throughout 2-year panels and those deceased during the panels, a total of 244,089 were used for PCA. Adjusting for survey design, the populations and the demographic characteristics were tabulated for the survey years in Table 1. With a weighting, the numbers of civilians were estimated to be 270 million in 1996 to 312 million in 2011, totaling 4.6 billion. The proportion of females (51.06% of the total) did not change significantly (*p* = 1). The mean ages increased from 34.69 to 37.20 (*p* < 0.001), while the proportion of whites (from 81.70 to 79.89%, *p* = 0.21) did not change significantly in the study period. The population by gender and age were plotted with the variances of the 525 PCs in Figure 2. Despite the decline of population numbers with the advanced age, the sum of variances of all PCs continued to increase after 45 years of age. In Figure 3, the proportions of explained variance by the first 20 PCs were shown. Most PCs did not explain more than 1% of total variances. The proportions explained by the first five PCs were as following: 48.24, 3.28, 2.72, 1.41, and 1.26%, respectively.

**Table 1:** Demographic characteristics of the populations in the Medical Expenditure Panel Surveys from 1996 to 2011.

Year	Unweighted sample sizes	Weighted sample sizes	(95% CI)	Mean Age	(95% CI)	Female	(95% CI)			
1996	19,526	270,220,553	(252,162,541–288,278,566)	34.56	(33.98–35.14)	51.14%	(50.44–51.84%)			
1997	12,242	272,924,582	(243,656,703–302,192,462)	34.65	(33.91–35.39)	51.12%	(50.29–51.94%)			
1998	9,956	275,276,754	(240,859,963–309,693,544)	34.98	(34.23–35.72)	51.38%	(50.42–52.34%)			
1999	13,120	277,777,364	(228,792,966–326,761,762)	34.90	(34.20–35.59)	51.27%	(50.52–52.02%)			
2000	10,237	282,678,581	(234,159,408–331,197,754)	35.36	(34.62–36.10)	51.21%	(50.22–52.19%)			
2001	20,823	287,502,824	(256,045,871–318,959,777)	35.54	(34.97–36.11)	51.17%	(50.57–51.78%)			
2002	15,911	289,917,944	(259,399,447–320,436,441)	35.62	(35.07–36.17)	51.02%	(50.26–51.78%)			
2003	15,972	292,395,934	(260,225,236–324,566,631)	35.80	(35.21–36.39)	50.99%	(50.36–51.62%)			
2004	15,839	295,173,059	(268,065,034–322,281,084)	36.02	(35.37–36.68)	51.07%	(50.31–51.83%)			
2005	15,388	298,082,232	(270,107,505–326,056,960)	36.07	(35.49–36.66)	50.91%	(50.14–51.68%)			
2006	16,260	299,880,574	(277,192,459–322,568,689)	36.20	(35.60–36.80)	50.93%	(50.27–51.59%)			
2007	12,237	302,814,992	(282,485,055–323,144,928)	36.62	(35.84–37.39)	51.03%	(50.26–51.79%)			
2008	17,995	305,990,159	(289,452,089–322,528,230)	36.39	(35.71–37.08)	50.79%	(50.05–51.53%)			
2009	15,993	307,472,984	(289,097,025–325,848,943)	36.61	(35.99–37.22)	50.85%	(50.16–51.54%)			
2010	14,338	310,617,388	(293,660,354–327,574,423)	37.02	(36.40–37.65)	51.17%	(50.36–51.97%)			
2011	18,252	312,413,749	(294,383,582–330,443,916)	37.19	(36.45–37.92)	51.09%	(50.40–51.78%)			
All	244,089	4,681,139,673	(4,502,416,344–4,859,863,002)	35.88	(35.67–36.09)	51.07%	(50.87–51.27%)			

**Year**	**White**	**(95% CI)**	**Black**	**(95% CI)**	**American Indians/Alaska natives**	**(95% CI)**	**Native Hawaiian/Pacific islanders**	**(95% CI)**	**Multiple races**	**(95% CI)**

1996	81.64%	(79.97–83.31%)	13.18%	(11.73–14.64%)	1.30%	(0.87–1.74%)	3.79%	(3.03–4.56%)		
1997	82.68%	(80.61–84.75%)	13.08%	(11.11–15.05%)	0.93%	(0.62–1.24%)	3.31%	(2.40–4.21%)		
1998	81.37%	(79.03–83.70%)	13.16%	(11.03–15.30%)	0.59%	(0.35–0.83%)	4.87%	(3.65–6.10%)		
1999	82.28%	(79.81–84.75%)	13.25%	(10.68–15.82%)	1.13%	(0.53–1.73%)	3.34%	(2.59–4.09%)		
2000	83.03%	(80.64–85.42%)	12.97%	(10.60–15.33%)	0.55%	(0.31–0.79%)	3.45%	(2.62–4.29%)		
2001	80.11%	(78.47–81.75%)	12.16%	(10.54–13.77%)	1.01%	(0.67–1.36%)	3.95%	(3.19–4.71%)	1.30%	(1.03–1.58%)
2002	81.40%	(79.83–82.96%)	12.34%	(10.87–13.81%)	0.79%	(0.53–1.05%)	3.94%	(3.15–4.72%)	0.27%	(0.14–0.40%)
2003	80.85%	(79.09–82.61%)	12.47%	(10.79–14.15%)	0.64%	(0.36–0.92%)	3.96%	(3.21–4.71%)	0.34%	(0.15–0.52%)
2004	80.28%	(78.34–82.23%)	12.44%	(10.68–14.19%)	0.81%	(0.48–1.14%)	4.25%	(3.39–5.12%)	0.37%	(0.16–0.57%)
2005	80.30%	(78.39–82.20%)	12.39%	(10.76–14.02%)	0.78%	(0.41–1.15%)	4.38%	(3.51–5.25%)	0.34%	(0.08–0.60%)
2006	80.18%	(78.48–81.87%)	12.41%	(11.02–13.80%)	0.91%	(0.52–1.30%)	4.32%	(3.48–5.16%)	0.44%	(0.19–0.70%)
2007	80.50%	(78.44–82.57%)	12.31%	(10.52–14.11%)	0.79%	(0.46–1.12%)	4.28%	(3.44–5.11%)	0.21%	(0.11–0.31%)
2008	79.87%	(78.08–81.66%)	12.43%	(11.02–13.84%)	0.78%	(0.41–1.15%)	4.54%	(3.71–5.37%)	0.25%	(0.11–0.40%)
2009	79.88%	(78.13–81.63%)	12.54%	(10.98–14.09%)	0.90%	(0.44–1.36%)	4.66%	(3.77–5.55%)	0.29%	(0.15–0.43%)
2010	79.56%	(77.57–81.56%)	12.29%	(10.74–13.84%)	0.81%	(0.30–1.32%)	4.96%	(3.92–5.99%)	0.68%	(0.36–1.00%)
2011	79.89%	(78.00–81.77%)	12.39%	(11.01–13.77%)	0.01%	–(0.01–0.03%)	5.14%	(4.00–6.29%)	0.01%	–(0.01–0.02%)
All	80.82%	(80.07–81.58%)	12.60%	(11.91–13.29%)	0.79%	(0.66–0.92%)	4.21%	(3.88–4.55%)	0.29%	(0.23–0.34%)

*The category of “multiple races” was introduced in 2001*.

*p < 0.001.

### Variables Contributing to the Variability of First PCs

In Tables 2–4, the variables were sorted by the contributed variance of the 40 leading variables for PC1 (first PC), PC2 (second PC), and PC3 (third PC). The leading variable, in terms of the variance contributed to PC1, was the amount spent on home health non-agency workers (hhnwcpy1, 99.3% variance contributed), followed by other measures on healthcare utilization (dvowcpy1, zidosry1, and many others) that included home care, dental care, emergency room use, prescriptions, and clinical visits (Table 2). There were 125 variables whose contributions to PC1 are >0.9 and 177 variables whose contributions are >0.8.

**Table 2:** The contribution of variance of each variable to PCs, sorted by the values in the first five PCs.

Variables	Labels	PC1	PC2	PC3	PC4	PC5
hhnwcpy1	HOME HLTH NON-AGNCY-WORKERS COMP AMT 11	0.993	0.003	0.000	0.000	0.000
dvowcpy1	ORTHODONTIST VISITS-WORKERS COMP AMT 11	0.993	0.003	0.000	0.000	0.000
zidosry1	ZERO-NITE IP STAZ-UNCLAS SRCE AMT-DR 11	0.992	0.003	0.000	0.000	0.000
hhnofdy1	HOME HLTH NON-AGNCY-OTHR FED AMT 11	0.992	0.003	0.000	0.000	0.000
obewcpy1	OPTOMETRIST OFF VSTS-WORKERS COMP AMT 11	0.991	0.003	0.000	0.000	0.000
zidvay1	ZERO-NITE IP STAZ-VA/CHAMPVA AMT-DR 11	0.991	0.003	0.000	0.000	0.000
zidstly1	ZERO-NITE IP STAZ-OTH ST/LOC AMT-DR 11	0.991	0.003	0.000	0.000	0.000
hhnopuy1	HOME HLTH NON-AGNCY-OTH PUBLIC AMT 11	0.991	0.003	0.000	0.000	0.000
obcopuy1	CHIRO OFF VISTS—OTHR PUBLIC AMT 11	0.991	0.003	0.000	0.000	0.000
dvomcry1	ORTHODONTIST VISITS—MEDICARE AMT 11	0.991	0.003	0.000	0.000	0.000
obcofdy1	CHIRO OFF VISITS—OTHER FEDERAL AMT 11	0.991	0.003	0.000	0.000	0.000
zifstly1	ZERO-NITE IP STAZ-OTH ST/LOC AMT-FAC 11	0.990	0.003	0.000	0.000	0.000
erdofdy1	ER-OTHER FED AMT—DR 11	0.990	0.003	0.000	0.000	0.000
dvoopuy1	ORTHODONTIST VISITS-OTHR PUBLIC AMT 11	0.990	0.003	0.000	0.000	0.000
zifopuy1	ZERO-NITE IP STAZ-OTH PUBLIC AMT-FAC 11	0.990	0.003	0.000	0.000	0.000
viswcpy1	GLASSES/CNTCT LENSES-WORKERS COMP AMT 11	0.990	0.003	0.000	0.000	0.000
dvoofdy1	ORTHODONTIST VISITS-OTHR FED AMT 11	0.990	0.003	0.000	0.000	0.000
dvovay1	ORTHODONTIST VISITS-VA/CHAMPVA AMT 11	0.990	0.003	0.000	0.000	0.000
zidopuy1	ZERO-NITE IP STAZ-OTH PUBLIC AMT-DR 11	0.989	0.003	0.000	0.000	0.000
zifofdy1	ZERO-NITE IP STAZ-OTHER FED AMT-FAC 11	0.989	0.003	0.000	0.000	0.000
obaopuy1	PHYS ASS T OFF VSTS—OTH PUBLIC AMT 11	0.989	0.003	0.000	0.000	0.000
opsofdy1	OPD DR VISITS-OTHER FED AMT-DR 11	0.989	0.003	0.000	0.000	0.000
hhnvay1	HOME HLTH NON-AGNCY-VA/CHAMPVA AMT 11	0.988	0.003	0.000	0.000	0.000
zifosry1	ZERO-NITE IP STAZ-UNCLAS SRCE AMT-FAC 11	0.988	0.003	0.000	0.000	0.000
opposry1	OPD NON-DR VSTS-OT UNCLAS SRC AMT-DR 11	0.988	0.003	0.000	0.000	0.000
obtopuy1	PT/OT OFF VISITS—OTH PUBLIC AMT 11	0.988	0.003	0.000	0.000	0.000
hhnosry1	H HLTH NON-AGNCY-OT UNCLASS SRCE AMT 11	0.988	0.003	0.000	0.000	0.000
obcstly1	CHIRO OFF VISITS—OTH ST/LOCAL AMT 11	0.987	0.003	0.000	0.000	0.000
obeopuy1	OPTOMETRIST OFF VSTS—OTH PUBLIC AMT 11	0.987	0.003	0.000	0.000	0.000
hhawcpy1	HOME HLTH AGENCY—WORKERS COMP AMT 11	0.987	0.003	0.000	0.000	0.000
oppstly1	OPD NON-DR VISITS-OTH ST/LOC AMT-DR 11	0.987	0.003	0.000	0.000	0.000
zidwcpy1	ZERO-NITE IP STAZ-WRKERS COMP AMT-DR 11	0.986	0.003	0.000	0.000	0.000
hhnopry1	HOME HLTH NON-AGNCY-OTH PRIVATE AMT 11	0.985	0.003	0.000	0.000	0.000
zifwcpy1	ZERO-NITE IP STAZ-WRKERS COMP AMT-FAC 11	0.985	0.003	0.000	0.000	0.000
oppofdy1	OPD NON-DR VISITS-OTHER FED AMT-DR 11	0.984	0.003	0.000	0.000	0.000
obeofdy1	OPTOMETRIST OFF VSTS-OTH FEDERAL AMT 11	0.984	0.003	0.000	0.000	0.000
othopuy1	OTHER EQUP/SUPPLY—OTH PUBLIC AMT 11	0.984	0.003	0.000	0.000	0.000
obtofdy1	PT/OT OFF VISITS—OTHER FED AMT 11	0.983	0.003	0.000	0.000	0.000
hhnstly1	HOME HLTH NON-AGNCY-OTHR ST/LOCL AMT 11	0.983	0.003	0.000	0.000	0.000
zifvay1	ZERO-NITE IP STAZ-VA/CHAMPVA AMT-FAC 11	0.982	0.003	0.000	0.000	0.000

*PC, principal component*.

*The numbers after the “.” in the variable names indicating the categories. Other variables not shown for limited space. The range of contribution of variable variance is from 0 (no variance contributed to PCs) to 1 (all of variable variance contributed)*.

**Table 3:** The contribution of variance of each variable to the second principal component, sorted by the contributed variance of variables in the second principal component.

Variables	Labels	PC1	PC2	PC3	PC4	PC5
pubjay1x.1	COVR BY ANY PUBLIC INS IN JAN11 (ED)	0.001	0.563	0.024	0.036	0.004
pubdey1x.1	COVR BY ANY PUBLIC INS IN DEC11 (ED)	0.001	0.540	0.028	0.044	0.002
mcrdey1x.1	COVERED BY MEDICARE IN DEC11 (ED)	0.017	0.516	0.035	0.006	0.002
obdmcry1	DR OFFICE VISITS—MEDICARE AMT 11	0.030	0.492	0.051	0.006	0.002
ssecpy1x	PERSON’S SOCIAL SECURITY INCOME 11	0.003	0.428	0.040	0.010	0.001
empst1.4	EMPLOYMENT STATUS RD 1	0.019	0.408	0.000	0.005	0.008
evrwrky1.1	EVER WRKD FOR PAY IN LIFE AS OF 12/31/11	0.016	0.391	0.021	0.003	0.005
actlim1.1	ANY LIMITATION WORK/HOUSEWRK/SCHL-RD 1	0.002	0.354	0.010	0.020	0.073
wlklim1.1	LIMITATION IN PHYSICAL FUNCTIONING-RD1	0.007	0.348	0.043	0.034	0.047
anylimy1.1	ANY LIMITATION IN P15R3,4,5/P16R1,2,3 11	0.025	0.348	0.052	0.043	0.034
pubdey1x.2	COVR BY ANY PUBLIC INS IN DEC11 (ED)	0.356	0.347	0.019	0.026	0.004
pubjay1x.2	COVR BY ANY PUBLIC INS IN JAN11 (ED)	0.357	0.330	0.022	0.028	0.004
inscovy1.2	HEALTH INSURANCE COVERAGE INDICATOR 11	0.005	0.323	0.170	0.038	0.018
fngrdf1	DIFFICULTY USING FINGERS TO GRASP-RD 1	0.123	0.299	0.034	0.031	0.055
mcrdey1x.2	COVERED BY MEDICARE IN DEC11 (ED)	0.410	0.293	0.021	0.005	0.000
unable1.1	COMPLETELY UNABLE TO DO ACTIVITY-RD 1	0.000	0.276	0.003	0.011	0.072
aidhlp1.1	USED ASSISTIVE DEVICES—RD 1	0.002	0.272	0.014	0.015	0.051
nwk1.2	REASON NOT WORKING DURING RD 1	0.016	0.246	0.041	0.000	0.014
iadlhp1.1	IADL SCREENER—RD 1	0.001	0.222	0.003	0.010	0.085
stjbyy1	YEAR STARTED RD 1 CMJ	0.101	0.222	0.131	0.160	0.057
anylimy1.2	ANY LIMITATION IN P15R3,4,5/P16R1,2,3 11	0.314	0.220	0.038	0.033	0.028
rxmcry1	TOTAL RX-MEDICARE AMT 11	0.087	0.217	0.012	0.004	0.000
evretiy1.1	PERSON HAS EVER RETIRED 11	0.018	0.214	0.059	0.002	0.008
iadlhp2.1	IADL SCREENER—RD 2	0.001	0.212	0.003	0.009	0.071
soclim1.1	SOCIAL LIMITATIONS—RD 1	0.002	0.207	0.009	0.013	0.059
rxtoty1	# PRESC MEDS INCL REFILLS 11	0.010	0.206	0.197	0.002	0.003
retpln1	PENSION PLAN AT RD 1 CMJ	0.015	0.199	0.089	0.160	0.059
retpln2	PENSION PLAN AT RD 2 CMJ	0.015	0.195	0.077	0.155	0.053
empst1.1	EMPLOYMENT STATUS RD 1	0.197	0.192	0.117	0.140	0.050
pegjay1.2	COVERED BY EMPL UNION INS IN JAN11	0.008	0.188	0.300	0.137	0.040
pegdey1.2	COVERED BY EMPL UNION INS IN DEC11	0.006	0.187	0.315	0.120	0.049
opfmcry1	ALL OPD VISITS-MEDICARE AMT-FAC 11	0.164	0.185	0.060	0.001	0.072
nwk1.3	REASON NOT WORKING DURING RD 1	0.000	0.182	0.001	0.006	0.031
obomcry1	NON-DR OFF VISTS—MEDICARE AMT 11	0.136	0.176	0.038	0.003	0.007
coglim1.1	COGNITIVE LIMITATIONS—RD 1	0.001	0.172	0.002	0.010	0.056
marryy1x.2	MARITAL STATUS-12/31/11 (EDITED/IMPUTED)	0.005	0.166	0.010	0.009	0.001
held2x.1	HEALTH INSUR HELD FROM RD 2 CMJ (ED)	0.105	0.145	0.223	0.000	0.177
ipfmcry1	IP HOSP STAZ-MEDICARE AMT-FAC 11	0.154	0.143	0.017	0.006	0.005
held1x.1	HEALTH INSUR HELD FROM RD 1 CMJ (ED)	0.103	0.141	0.230	0.001	0.177
totmcdy1	TOTAL AMT PAID BY MEDICAID 11	0.112	0.140	0.107	0.095	0.042

*PC, principal component*.

*The numbers after the “.” in the variable names indicating the categories. Other variables not shown for limited space. The range of contribution of variable variance is from 0 (no variance contributed to PCs) to 1 (all of variable variance contributed)*.

**Table 4:** The contribution of variance of each variable to the third principal component, sorted by the contributed variance of variables in the third principal component.

Variables	Labels	PC1	PC2	PC3	PC4	PC5
totprvy1	TOTAL AMT PAID BY PRIVATE INS 11	0.162	0.028	0.420	0.092	0.000
obdprvy1	DR OFFICE VISITS—PRIVATE INS AMT 11	0.068	0.010	0.334	0.078	0.002
rxprvy1	TOTAL RX-PRIVATE INS AMT 11	0.029	0.008	0.331	0.032	0.001
hpejay1.1	HOLDER OF EMPL UNION INS IN JAN11	0.120	0.071	0.318	0.002	0.159
pegdey1.2	COVERED BY EMPL UNION INS IN DEC11	0.006	0.187	0.315	0.120	0.049
hpedey1.1	HOLDER OF EMPL UNION INS IN DEC11	0.125	0.082	0.304	0.001	0.159
pegjay1.2	COVERED BY EMPL UNION INS IN JAN11	0.008	0.188	0.300	0.137	0.040
hpedey1.2	HOLDER OF EMPL UNION INS IN DEC11	0.116	0.086	0.293	0.000	0.175
hpejay1.2	HOLDER OF EMPL UNION INS IN JAN11	0.120	0.088	0.274	0.004	0.156
opfprvy1	ALL OPD VISITS-PRIV INS AMT-FAC 11	0.027	0.008	0.271	0.019	0.219
pegjay1.1	COVERED BY EMPL UNION INS IN JAN11	0.273	0.118	0.248	0.096	0.028
totslfy1	TOTAL AMT PAID BY SELF/FAMILY 11	0.219	0.046	0.234	0.000	0.002
pegdey1.1	COVERED BY EMPL UNION INS IN DEC11	0.282	0.132	0.234	0.096	0.028
held1x.1	HEALTH INSUR HELD FROM RD 1 CMJ (ED)	0.103	0.141	0.230	0.001	0.177
oboprvy1	NON-DR OFF VISTS—PRIVATE INS AMT 11	0.000	0.000	0.225	0.016	0.003
held2x.1	HEALTH INSUR HELD FROM RD 2 CMJ (ED)	0.105	0.145	0.223	0.000	0.177
opdprvy1	ALL OPD VISITS-PRIV INS AMT-DR 11	0.085	0.007	0.211	0.014	0.226
obdslfy1	DR OFFICE VISITS—SELF/FAMILY AMT 11	0.053	0.001	0.204	0.006	0.011
mcddey1.1	COV BY MEDICAID OR SCHIP IN DEC11	0.016	0.111	0.201	0.144	0.030
opdexpy1	TOTAL OUTPATIENT PROVIDER EXP 11	0.053	0.066	0.201	0.006	0.275
rxtoty1	# PRESC MEDS INCL REFILLS 11	0.010	0.206	0.197	0.002	0.003
pridey1	COVERED BY PRIVATE INS IN DEC11	0.287	0.071	0.193	0.054	0.018
mcdjay1.1	COV BY MEDICAID OR SCHIP IN JAN11	0.015	0.107	0.185	0.133	0.032
marryy1x.6	MARITAL STATUS-12/31/11 (EDITED/IMPUTED)	0.004	0.003	0.183	0.331	0.035
inscovy1.2	HEALTH INSURANCE COVERAGE INDICATOR 11	0.005	0.323	0.170	0.038	0.018
optotvy1	# OUTPATIENT DEPT PROVIDER VISITS 11	0.259	0.076	0.168	0.003	0.167
opoprvy1	OPD NON-DR VISITS-PRIV INS AMT-FAC 11	0.087	0.005	0.166	0.009	0.118
intrpy1x	PERSON’S INTEREST INCOME 11	0.014	0.007	0.165	0.004	0.000
opoexpy1	TOTAL OUTPATIENT NON-DR—FAC EXP 11	0.049	0.063	0.159	0.002	0.141
wrglas2.2	WEARS EYEGLASSES OR CONTACTS—RD 2	0.054	0.042	0.158	0.020	0.007
obothvy1	# OFFICE-BASED NON-PHYSICAN VISITS 11	0.041	0.035	0.158	0.001	0.004
opvprvy1	OPD DR VISITS-PRIV INS AMT-FAC 11	0.088	0.003	0.147	0.012	0.149
wrglas2.1	WEARS EYEGLASSES OR CONTACTS—RD 2	0.162	0.046	0.143	0.017	0.004
oboslfy1	NON-DR OFF VISTS—SELF/FAMILY AMT 11	0.002	0.001	0.136	0.000	0.007
obdtchy1	OFFICE-BASED PHYSICIAN VISIT CHARGES 11	0.099	0.109	0.136	0.044	0.000
coglim1.2	COGNITIVE LIMITATIONS—RD 1	0.305	0.002	0.134	0.194	0.007
stjbyy1	YEAR STARTED RD 1 CMJ	0.101	0.222	0.131	0.160	0.057
taxfrmy1.1	TAX FORM PERSON WILL FILE 11	0.078	0.003	0.131	0.012	0.002
insjay1x.2	COVR BY HOSP/MED INS IN JAN11 (ED)	0.003	0.014	0.128	0.390	0.067
insdey1x.2	COVR BY HOSP/MED INS IN DEC11 (ED)	0.003	0.015	0.127	0.393	0.074

*PC, principal component*.

*The numbers after the “.” in the variable names indicating the categories. Other variables not shown for limited space. The range of contribution of variable variance is from 0 (no variance contributed to PCs) to 1 (all of variable variance contributed)*.

The leading variables, in terms of the contributed variances to PC2, were public insurance coverage (pubjay1x.1, pubdey1x.1, and inscovy1.2), employment-related variables (empst1.4), and functional limitations (actlim1.1, wlklim1.1, anylimy1.1, Table 3). However, there were only three variables contributing more than 0.5 to PC2. In Table 4, there were no variables contributing more than 50% of their own variances to PC3. The leading variables for PC3 were related to the amount of health-care expenditures paid by private insurance and coverage of employment-related insurance. For the other PCs, the contribution of the leading variable decreased.

### Life Stages

**Figure 4:**
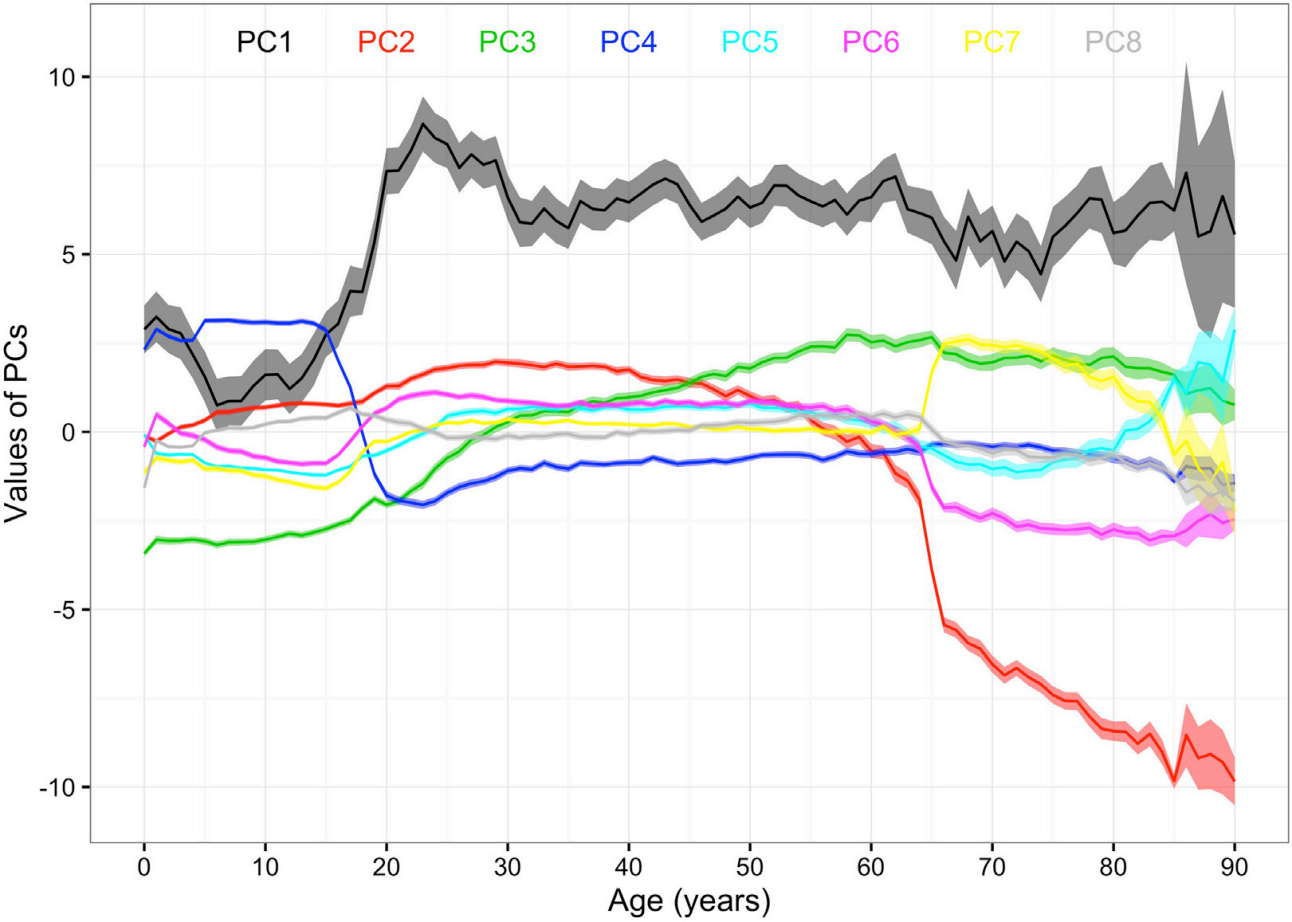
Mean values of the first to the eighth principal components (PCs) by age. Note: 95% CIs of the PCs are the ranges of the same colors as the PCs. The colors and corresponding numbers of PCs are labeled on the graph.

**Figure 5:**
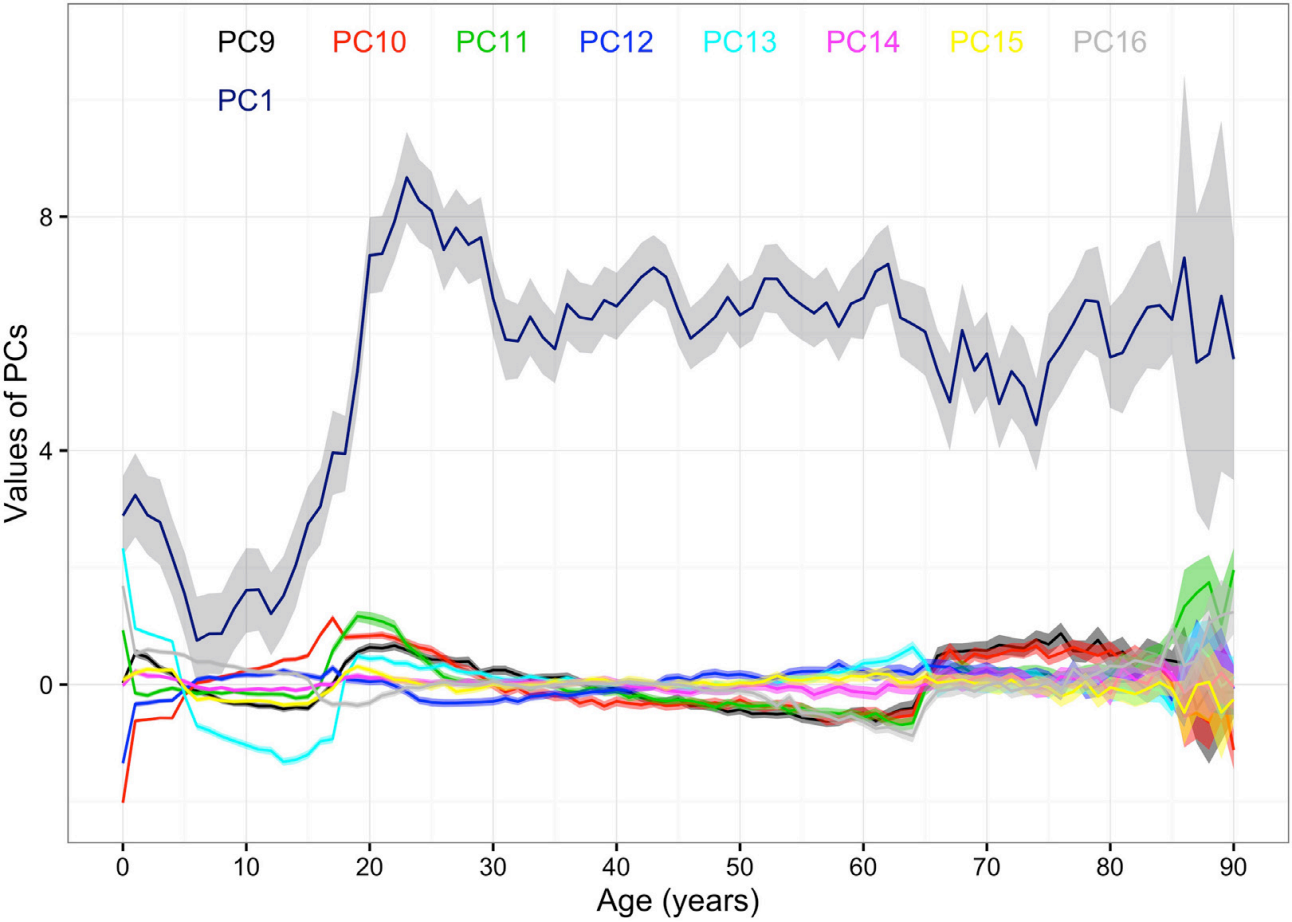
Mean values of the 1st and the 9th–16th principal components (PCs) by age. Note: 95% CIs of the PCs are the ranges of the same colors as the PCs. The color of first PC is navy blue. The colors and corresponding numbers of PCs are labeled on the graph.

**Figure 6:**
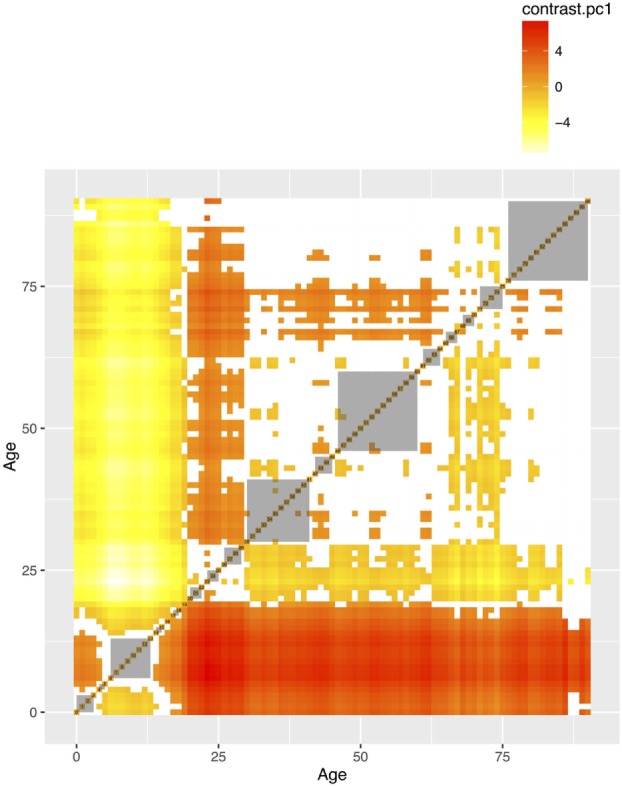
The pairwise comparisons of the first principal component by age groups. The value in each cell is the difference of PC1 between two age groups. The differences that are not statistically significant (*p* values adjusted for multiple comparisons based on the Benjamini–Hochberg method at 0.05) were left blank. The gray areas are the groups of consecutive ages with less than 5% significant differences in pairwise comparisons.

**Figure 7:**
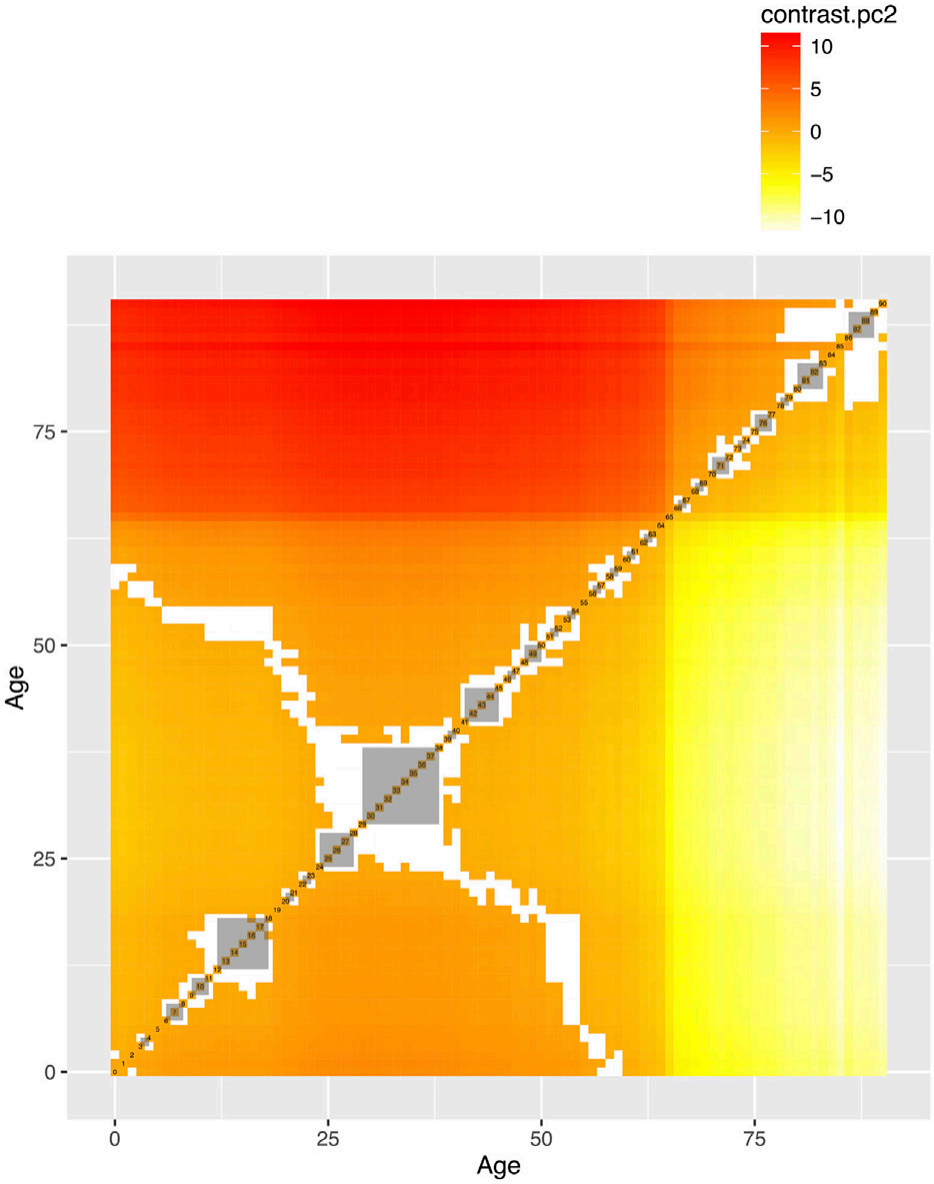
The pairwise comparisons of the second principal component (PCs) by age groups. The value in each cell is the difference of PC1 between two age groups. The differences that are not statistically significant (*p* values adjusted for multiple comparisons based on the Benjamini–Hochberg method at 0.05) were left blank. The gray areas are the groups of consecutive ages with less than 5% significant differences in pairwise comparisons.

The mean values of the first 16 PCs were plotted in Figures 4 and 5. The first eight PCs had changes of greater magnitude than the 9–16th PCs between age of 0 and 90 years. To test the significance of the changes in PC values, Figures 6 and 7 present the pairwise comparisons of the PC1 and PC2 by age, respectively. By setting the insignificant values to blank in the matrix of differences, there were clusters of blank cells along with the diagonal axis that labeled years of age. In the gray rectangles, the covered areas had less than 5% of significant differences of all cells in the areas. Therefore, the age ranges covered by gray rectangles were stages with populations of similar PC values. The values of PC1 were not different from 6 to 13, 30 to 41, 46 to 60, and 71 to 90 (adjusted *p* values > 0.05 for more than 95% of all pairwise comparisons). The values of PC2 were not different from 12 to 18, 29 to 38, and 41 to 45 years.

### Major Determinants of PCs

To have a good approximation of PC1 and PC2 (*R*^2^ > 0.8), it required 13 and 41 variables (see Table 5 for a partial list). The leading variables that explained the most of the PC1 variance were income and poverty status, functional and cognitive limitations, marital status, and perceived health status. In addition to marital status, cognitive limitations and income, social security income, clinical visits and healthcare expenditures covered by Medicare, and employment status were the leading variables explaining PC2. The life stages identified with the first two PCs, especially PC1, were associated with the statuses of marital status, income and cognitive limitations.

**Table 5:** R squared of the leading variables that best explained first principal component.

Variables	Labels	PC1	PC2	PC3	PC4
wcmppy1x	PERSON’S WORKERS’ COMPENSATION 11	0.247	0.056	0.101	0.013
povcaty1	FAMLY INC AS % OF POVERTY LINE-CATEGO 11	0.243	0.017	0.032	0.002
marryy1x.5	MARITAL STATUS-12/31/11 (EDITED/IMPUTED)	0.110	0.003	0.061	0.017
marryy1x.3	MARITAL STATUS-12/31/11 (EDITED/IMPUTED)	0.049	0.012	0.004	0.015
actlim1.2	ANY LIMITATION WORK/HOUSEWRK/SCHL-RD 1	0.033	0.020	0.015	0.002
coglim1.2	COGNITIVE LIMITATIONS - RD 1	0.026	0.003	0.001	0.012
rthlth1	PERCEIVED HEALTH STATUS - RD 1	0.022	0.006	0.018	0.011
intrpy1x	PERSON’S INTEREST INCOME 11	0.019	0.009	0.016	0.002
totslfy1	TOTAL AMT PAID BY SELF/FAMILY 11	0.019	0.013	0.004	0.000
coglim1.1	COGNITIVE LIMITATIONS - RD 1	0.016	0.040	0.003	0.003

*PC, principal component*.

*The numbers after the “.” in the variable names indicating the categories. Other variables not shown for limited space. The range of R squared of the input variable is from 0 (no variance of the PCs explained by input variables) to 1 (all of variance of the PCs explained by input variables)*.

## Discussion

### Research Implications

This study showed that a complex dataset like the MEPS could be summarized and life stages could be adaptively identified from the data, based on the explicit criteria and statistical tests. There are several research implications. First, this is the first attempt to systematically assess the data from all age groups to better define and identify stages of stability or transition. This method can be applied to other data sets to construct a systematic process and to obtain an insight from the stages of stability and transition.

Second, the identification of life stages is important for research that relies on a research population with similar characteristics to draw samples for follow-up or intervention (33, 34). The gray rectangles in Figures 6 and 7 show that: civilians from the same stable life stages could be more comparable than those in transition stages, in terms of the PC values. Third, these life stages are also important for epidemiological investigation that sometimes a population stratification is needed to augment sample sizes in each age category (35). The beginning and the end ages of life stages could serve as references for the age stratification, if there is no empirical evidence or prior knowledge about the population under investigation. Our results provide an alternative choice of selecting age cutoffs, especially for those who lack empirical evidence to stratify age groups or information on the stages of life in social and health data.

Fourth, the application of life staging might be an important method to understand the sources of variability in the dataset, while PCA is recommended as the first step to explore the information observed, especially for complex data (36). This is increasingly important while we observe that there are many variables contributing high proportions of their own variances to PC1, but might not be frequently used for empirical research (see Tables 2–5 for the leading variables). This highlights a potential problem of information underuse and a lack of comprehensive understanding of the life course in aging and social data. This problem is particularly acute when many researchers adopt a uni-dimensional view of trajectories across certain stages of life (3, 6, 37) or propose multiple trajectories without summarizing them (38). This study aims to be the first to call attention to the potential of underused measurements in existing surveys.

Fifth, this data-driven approach also identified that the first PC seem to be related to and can be approximated with similar input variables, such as health insurance categories. This is useful for epidemiologists or data scientists, who aim to construct indexes that explain a large portion of the overall variance in the data set. We showed that this line of research may be feasible with major national surveys. We are developing this method as a strategy to systematically understand large datasets. Sixth, certain life stages might be important for the future research to understand the cause of decline or incline across a life course, such as the stages of transition identified with PC1 and PC2 values. We will focus on the life stages with significant transitions and search for a possible mechanism of these fluctuations.

### Limitations

There are several limitations to this study. First, the MEPS dataset is implemented in the US with a focus on the health coverage and related issues (16). Other datasets or surveys may focus on other topics, concentrate on certain age groups or are created in distinctive manners, such as in other jurisdictions or under different policies. Individual health coverage and health-care utilization could also be influenced by public policies. For example, the Affordable Care Act sought to increase health coverage and improve access to health care (39). Therefore, the PCA analysis results with the MEPS dataset may not be able to be extrapolated.

The second limitation is also related to the contexts. The detailed data on life stages help us to have a better understanding of the ages of transitions across life course. However, the PCA results are influenced by how and what data are collected in different age groups. The sum of all variances between individuals depends on the variable variances that are directly influenced by the numbers of measures on similar characteristics and measurement scales (continuous or categorical). For example, the questionnaire of the Children with Special Health Care Needs in the MEPS is applicable to those ages of 0–17 years, although not selected for this study (22). In addition, the MEPS only surveys non-institutionalized civilians and it is unclear how this may affect the results. By recognizing these limitations, caution would be necessary for those life stages identified due to questionnaire changes, rather than transitions in the life course.

Third, there might be some questionnaire modifications introduced between 1996 and 2011 that we are not aware of. We have read through various documentation to accommodate modifications in racial categories and other variable definitions (22). However, we cannot guarantee that all questionnaire revisions are reflected in the data processing. Fourth, the cross-sectional nature of the MEPS makes it possible to have data on individuals from the whole age spectrum and illustrate trajectories across life course. However, individual trajectories are followed-up for only 2 years. It remains uncertain whether the population trajectories of mean PC values can reflect individual ones.

Fifth, the trajectories might be partly caused by the social or health policies that aim to improve population literacy or income security, such as compulsory education for children and retirement arrangements before 65 years of age. This confounding factor needs a separate study. Sixth, adjusting the complex survey design is important for maintaining national representativeness, but this leaves fewer choices of analytical tools. For example, to the best of our knowledge, there is only linear PCA available for datasets with complex survey design and no tool to implement the correlation-based feature selection process after taking the survey design into account (25). Non-linear or other types of PCA that some researchers propose for categorical variables are also not applicable for data with survey design (27, 40). Finally, there remains room for debate on what measures should or could be used to determine life stages. The use of PCs to determine life stages with the social and health data may not be optimal for researchers who need biological or epigenetic measures (41–43).

### Future Work

The abovementioned limitations also suggest more research opportunities that we will explore later. The first of our future research directions will be to demonstrate the use of each PC to other datasets and produce components that can be generalized to future panels of MEPS data and other data sources. The second research opportunity will be to demonstrate the usefulness of the concept of life stages. The priority is to apply this research framework on other longitudinal data sets as the benchmark, such as the Health and Retirement Study (44) that has been widely used in the research community. Third, we will use biomarker database to demonstrate the life stages based on PCs. The fourth opportunity will be the selection of the information sources in specific life stages with different age groups. This aims to partly solve the issues related to questionnaire changes for specific age groups. Fifth, the life stages based on chronological ages may not be optimal, since the biological clock or DNA methylation age may differ to some extent (41). It may be more useful to link the biological ages with the observed life stages.

## Conclusion

This study showed that complex datasets like MEPS could be summarized to identify life stages and their major determinants, including the statuses of functionality and cognition, income, and marital status. The identification of stable and transition life stages is important for research that relies on a research population with similar characteristics to draw samples for observation or intervention. There are research opportunities regarding the periods of transitions and the causes of different trajectories.

## Patient Consent

The MEPS data are publicly available, and there is no patient consent form available for download.

## Availability of Data and Materials

All data sets can be freely assessed *via* the Agency for Healthcare Research and Quality website (https://meps.ahrq.gov/data_stats/download_data_files.jsp).

## Ethics Statement

This secondary data analysis study was approved by the ethics committee of the Centre hospitalier de l’Université de Montréal (number: 2016-6095).

## Author Contributions

Y-SC conceptualized the research project, restructured the data, conducted the statistical analyses, and drafted the manuscripts. H-TW provided suggestions from different aspects and revised the manuscript. C-JW reviewed the manuscript and provided constructive comments.

## Conflict of Interest Statement

The authors declare that the research was conducted in the absence of any commercial or financial relationships that could be construed as a potential conflict of interest.

## Abbreviations

MEPS, Medical Expenditure Panel Survey; PCA, principal component analysis; ADL, activities of daily living; IADL, instrumental activities of daily living; PHQ-2, Patient Health Questionnaire; PC, principal component; PC1, the principal component explaining the most portion of total variance; PC2, the principal component explaining the second most portion of total variance.
